# Establishing Standards for Studying Renal Function in Mice through Measurements of Body Size-Adjusted Creatinine and Urea Levels

**DOI:** 10.1155/2014/872827

**Published:** 2014-08-27

**Authors:** Wellington Francisco Rodrigues, Camila Botelho Miguel, Marcelo Henrique Napimoga, Carlo Jose Freire Oliveira, Javier Emilio Lazo-Chica

**Affiliations:** ^1^Postgraduate Course in Health Sciences, Federal University of Triângulo Mineiro, 38061-500 Uberaba, MG, Brazil; ^2^Cefores, Federal University of Triangulo Mineiro, 38015-050 Uberaba, MG, Brazil; ^3^Laboratory of Immunology and Molecular Biology, São Leopoldo Mandic Institute and Research Center, 13045-755 Campinas, SP, Brazil; ^4^Institute of Biological and Natural Sciences, Federal University of Triângulo Mineiro, 38015-050 Uberaba, MG, Brazil

## Abstract

Strategies for obtaining reliable results are increasingly implemented in order to reduce errors in the analysis of human and veterinary samples; however, further data are required for murine samples. Here, we determined an average factor from the murine body surface area for the calculation of biochemical renal parameters, assessed the effects of storage and freeze-thawing of C57BL/6 mouse samples on plasmatic and urinary urea, and evaluated the effects of using two different urea-measurement techniques. After obtaining 24 h urine samples, blood was collected, and body weight and length were established. The samples were evaluated after collection or stored at −20°C and −70°C. At different time points (0, 4, and 90 days), these samples were thawed, the creatinine and/or urea concentrations were analyzed, and samples were restored at these temperatures for further measurements. We show that creatinine clearance measurements should be adjusted according to the body surface area, which was calculated based on the weight and length of the animal. Repeated freeze-thawing cycles negatively affected the urea concentration; the urea concentration was more reproducible when using the modified Berthelot reaction rather than the ultraviolet method. Our findings will facilitate standardization and optimization of methodology as well as understanding of renal and other biochemical data obtained from mice.

## 1. Introduction

The kidneys have homeostatic, regulatory, and excretory roles and, depending on their ability to function, they can exhibit a variety of pathological conditions. However, more than a century after the first development of an assay for evaluating kidney function [1], some doubts persist about the correct method for evaluation of the function of this organ.

Among the current tools used to evaluate renal function, biochemical measurements are the most commonly used and include evaluation of creatinine clearance, by analyzing the serum, plasma, and urine creatinine levels and of urea concentration, by assessing the plasma concentration and excretion of urea [2]. The main techniques used to evaluate serum, plasma, and urine creatinine levels are the colorimetric assay, employing alkaline picrate [1], and enzymatic assays [3], while high-performance liquid chromatography (HPLC) is also used to a lesser extent [4, 5]. To assess urea concentration, colorimetric and enzymatic assays are also used; the vast majority of these employ an enzyme that breaks down urea (urease) as well as a coupled enzyme that uses ammonia as substrate.

The methods currently used for evaluating creatinine clearance have been compared in a study investigating the sera of different animal species; these methods vary significantly in their measurement of creatinine clearance in serum and overestimate or underestimate the actual glomerular filtration rate, which is represented by the clearance [6]. Besides these analytical variations, another important factor influencing measurement of creatinine clearance is the ratio of weight to height, which determines body surface area (BSA); creatinine clearance is typically corrected for the latter. Although the methodological parameters of height and weight for determining BSA have been determined in humans, these values cannot be applied for assessment of creatinine clearance in experimental animal models, such as mice.

With regard to laboratory methods for measurement of urea, the techniques commonly used do not suffer from analytical variation; however, these techniques are often used to assess samples collected at various temperatures and stored for different periods of time [7, 8], and, as already shown for canine and equine species, these variations in time and temperature may lead to different estimations of urea concentrations. Furthermore, although there are no significant discrepancies between the techniques, the variation of standard deviation in duplicate or triplicate in acquiring the mean values should be considered.

Thus, given that there may be variations in both the laboratory techniques and the methods used for determining creatinine clearance and urea concentration, the objective of this study was to determine an average factor from the murine BSA for the calculation of biochemical renal parameters, to determine the effects of storage and freeze-thawing of mice serum on plasmatic and urinary urea measurements and to evaluate the effects of using two different urea-measurement techniques that are in common laboratory use.

## 2. Methods

### 2.1. Animals

All experiments were conducted in accordance with the National Health guidelines for the welfare of experimental animals and with the approval of the Ethical Committee of the University Federal of Triângulo Mineiro (# 150/2010). Adult male C57BL/6 mice (*n* = 40) weighing 19–25 g, and aged 8–12 weeks, were housed in temperature-controlled rooms (22–25°C), with access to water and food ad libitum.

### 2.2. Biological Samples

After obtaining 24 h urine samples from the mice in a metabolic cage, the animals were fasted for 4 h; after this procedure the animals were heparinized (40 units of Hemofol, 5000 IU/mL) and euthanized in a CO_2_ chamber. Then, blood was removed through the ophthalmic plexus with the aid of a glass Pasteur pipette. The blood was subjected to centrifugation at 1831 ×g for 10 min to obtain plasma.

### 2.3. Quality Control

We implemented internal quality control processes, where a clear definition of objectives, procedures, standards, and criteria for the tolerance limits, corrective actions, and registration of the activities, as well as the use of controls to evaluate the imprecision of the analysis, were stated and monitored. Control charts, that is, those of Levey-Jennings and the multiple Rules of Westgard were also implemented [9, 10].

### 2.4. Ratio of Weight and Height for Determining BSA

For the standardization of BSA, we used the following equation: BSA = weight (*W*)^0.425^ × length (*L*)^0.725^ × 0.007184 [11].

### 2.5. Creatinine Clearance

The amount of creatinine in plasma and 24 h urine was determined in nine animals using a commercial kit (Biotechnical; Varginha, Minas Gerais, Brazil) based on a kinetic (two-point) colorimetric method (red-yellow) that employs picrate in an alkaline solution. Absorbance measurements were performed using a semiautomated method, using a spectrophotometer (Bioplus-2000 Barueri, São Paulo, Brazil) at a wavelength of 500 nm and a water bath at 37°C (Sieger-Stern 6, Campo Mourão, Paraná, Brazil), without diluting or concentrating the samples. Creatinine clearance was expressed in mL/min, obtained by the following equation:

(1) Clearance (mL/min⁡/XmBSm2) =(D [mL/min⁡]×XmBS2)BS,

where *D* = depuration, which is equal to the concentration of urine creatinine (mg/dL)/concentration of plasma creatinine (mg/dL) × urinary volume in 24 h (mL), and XmBSm^2^ = ∑*n*BS/*n*, where *n* = number of animals, and BS = body surface area.

### 2.6. Variables for Determination of Analytical Variations in the Concentration of Urea

To evaluate analytical changes in the quantification of urea, we performed three experiments.

1. Plasma samples from two separate sets of experiments (*n* = 10 animals) were subjected to freezing (on the day of collection and on the 4th day after collection) and thawing (on the 4th and 90th days after collection); thereafter, the urea concentration was measured, without varying the temperature.
2. Plasma and urine (*n* = 10 animals) were collected and immediately separated into 12 different aliquots to allow quantifying urea concentration after different periods of storage (0, 4, and 90 days) and different storage temperatures (room temperature, −20°C and −70°C).
3. The concentration of urea from urine samples was determined using two different methods (enzymatic colorimetric and ultraviolet), without storage period or temperature variations.

### 2.7. Urea

Plasma and urinary urea were quantified using commercial kits (urea ultraviolet and the modified Berthelot reaction; Biotechnical, Varginha, Minas Gerais, Brazil). The absorbance readings were obtained using a semiautomated method, employing a spectrophotometer (Bioplus-2000 Barueri, São Paulo, Brazil) at wavelengths of 340 and 580 nm, respectively, and using a water bath at 37°C (Sieger-Stern 6, Campo Mourão, Paraná, Brazil). All parameters of quality control were followed as described above.

### 2.8. Statistical Analysis

Statistical analysis was performed using Prism 4.0 software (GraphPad, La Jolla, CA, USA). The data were fist examined for nomality and comparison of the variances (Kolmogorov–Smirnov test or *F*-test). When the distribution was Gaussian the data were analyzed using a parametric test (paired *t*-test for two events, and Repeated Measures ANOVA with Tukey post-test for more than two events). In cases where the distribution was not Gaussian, nonparametric tests (Friedman test with Dunn's post-test for more than two events, and Wilcoxon matched pairs test for two events). Mann-Whitney test was used to test for differences between the duplicates by comparing the two methods, because the distribution was not Gaussian. The differences were considered significant when *P* < 0.05.

## 3. Results

Length and weight were measured in all 40 C57BL/6 mice at the start of the experiments. The mean ± standard deviation of weight and length is presented in Table 1. The BSA of each animal was calculated and an average factor (0.006179) was obtained, which was used for determination of creatinine clearance.

As shown in Table 2, the concentration of creatinine in plasma and urine and the volume of urine excreted in a period of 24 h varied markedly between the maximum and minimum levels, although not statistically significantly (*P* > 0.05).

A detailed standard procedure, which aimed to incorporate measures for internal quality control, such as acceptance adjustment or invalidation, was employed. Thus, we validated our analysis using the multirules of Westgard [10].

**Figure 1:**
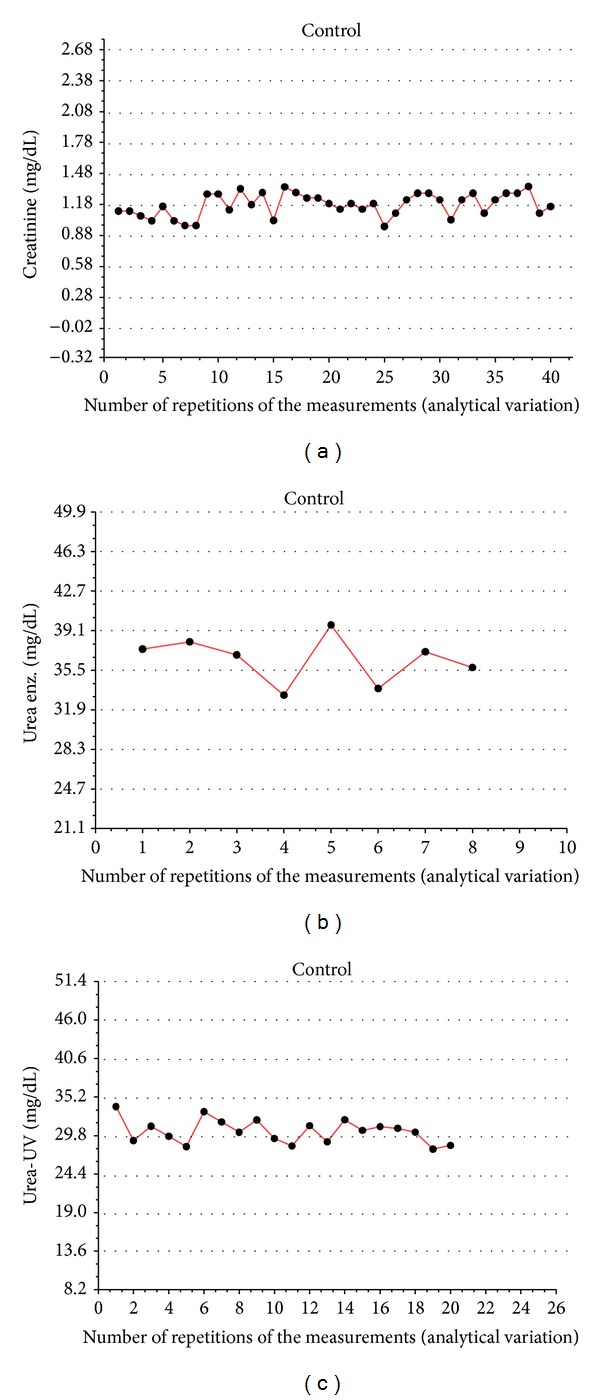
Levey-Jennings charts and evaluation of Westgard rules. The control samples were obtained from the biotechnical company and the variations (mean and standard deviation) were established and compared with those of the manufacturer, obtained from 20 different trials. After the collection and preparation of all biological samples, we inserted the control samples among the test samples, randomly, and quantified the concentrations of urea and creatinine by spectrophotometry. In (a), we evaluated the concentrations of creatinine by the colorimetric method (red-yellow), using picrate in an alkaline solution and reading at 500 nm, using 40 control points with an average of 1.18 and a 0.30 to 1 s deviation. In (b), we evaluated the concentrations of urea by the modified method of Berthelot (reading at 580 nm), using eight control points, with an average of 35.5 and a deviation of 3.60 to 1 s. In (c), we evaluated the concentrations of urea spectrophotometrically at 340 nm, using 20 control points, with an average of 29.8 and a deviation of 5.40 to 1 s. For statistical evaluation, we used the deviation distribution of samples (1 s, 2 s, and 3 s) as well as the Westgard rules.

As depicted in Figure 1, none of the rules proposed by Westgard were violated for any of the parameters evaluated. The measurements of all 40 animals tested showed small variations in creatinine concentrations (Figure 1(a)), but the data fell within the average and standard deviation (mean [SD]: 1.18 [0.30] mg/dL). As shown in Figure 1(b), quantification of the concentrations of urea by the enzymatic colorimetric method (580 nm; mean [SD]: 35.5 [3.6] mg/dL) was performed with eight repetitions. For the analysis of urea concentration (Figure 1(c)), 20 plasma samples were quantified using the UV urea method (340 nm; mean [SD]: 29.8 [5.4] mg/dL) and the values fell within the average and standard deviation. Thus, altogether these data allowed us to assume that the results were within the limits of acceptance as described in the Westgard multirules [10].

**Figure 2:**
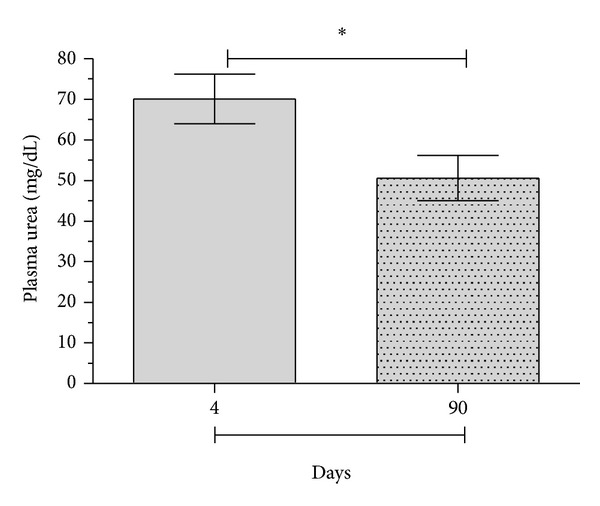
Influence of thawing on the measurement of plasma urea concentration. Blood was obtained from C57BL/6 mice for measurement of plasma urea. After centrifugation, the plasma was separated into tubes and frozen at −20°C for 4 days, followed by thawing and measurement of plasma urea by spectrophotometry. Samples were then again frozen at −20°C for 86 more days, when plasma urea was again evaluated. Values are expressed in mg/dL; (∗) statistically significant differences at *P* < 0.05.

In order to evaluate the effect of freeze-thawing on plasma urea concentration, the plasma samples were frozen at −20°C and thawed after 4 or 90 days after collection. As demonstrated in Figure 2, there was a significant decrease (*P* < 0.05) in the urea concentration of the thawed samples by 90 days after collection, as compared with the same samples thawed only 4 days after collection. This result demonstrated the importance of standardization and careful handling of biological samples in facilitating reliable determination of urea concentration.

**Figure 3:**
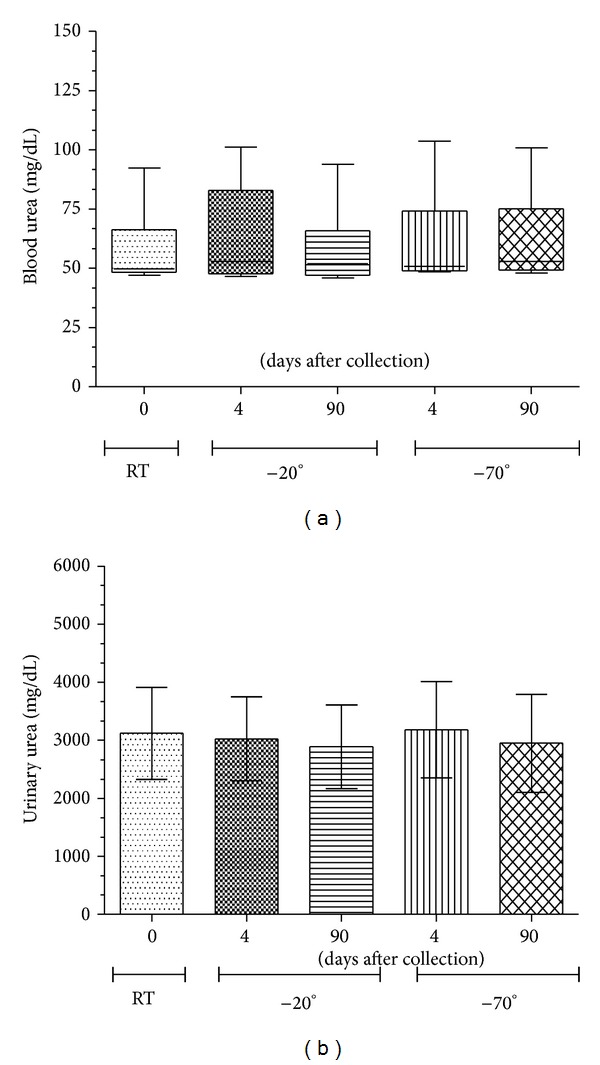
Influence of freezing on the quantification of plasma and urinary urea. Blood and urine were obtained from C57BL/6 mice and urea concentration was determined. Urine was obtained from mice maintained in metabolic cages for a period of 24 h. After urine collection, the blood was collected from the ophthalmic plexus. Both types of samples were centrifuged at 1831 ×g for 10 min and supernatants were divided into different aliquots. An initial evaluation of the samples was made at room temperature, before freezing, and further aliquots were frozen at −20°C or −70°C for 4 or 90 days, followed by quantification of the respective blood or urinary urea. In (a), we evaluated the concentrations of plasma urea by the colorimetric method (ultraviolet), following the time and temperature variations described above. In (b), we evaluated the concentrations of urinary urea at 24 h, using the same colorimetric method (ultraviolet) as for the plasma. Values are expressed in mg/dL. No significant differences at *P* < 0.05 were observed.

After we investigated the variation in the analytical concentrations of urea with differences in the storage period, new measurements of the urea concentrations were made for different storage periods and storage temperatures. For this evaluation, samples were not frozen and thawed more than once, and we evaluated the samples immediately after collection (at room temperature). As shown in Figure 3, there were no significant differences in the urea concentration of blood or urine samples evaluated after different storage periods (0, 4, or 90 days after collection) or for different storage temperatures (room temperature, −20 or −70°C). Therefore, freezing is an important method of preservation, although standardization of standard operating procedures is still required in order to contribute to the reliability of the measurements, taking into consideration the analyte to be quantified.

**Figure 4:**
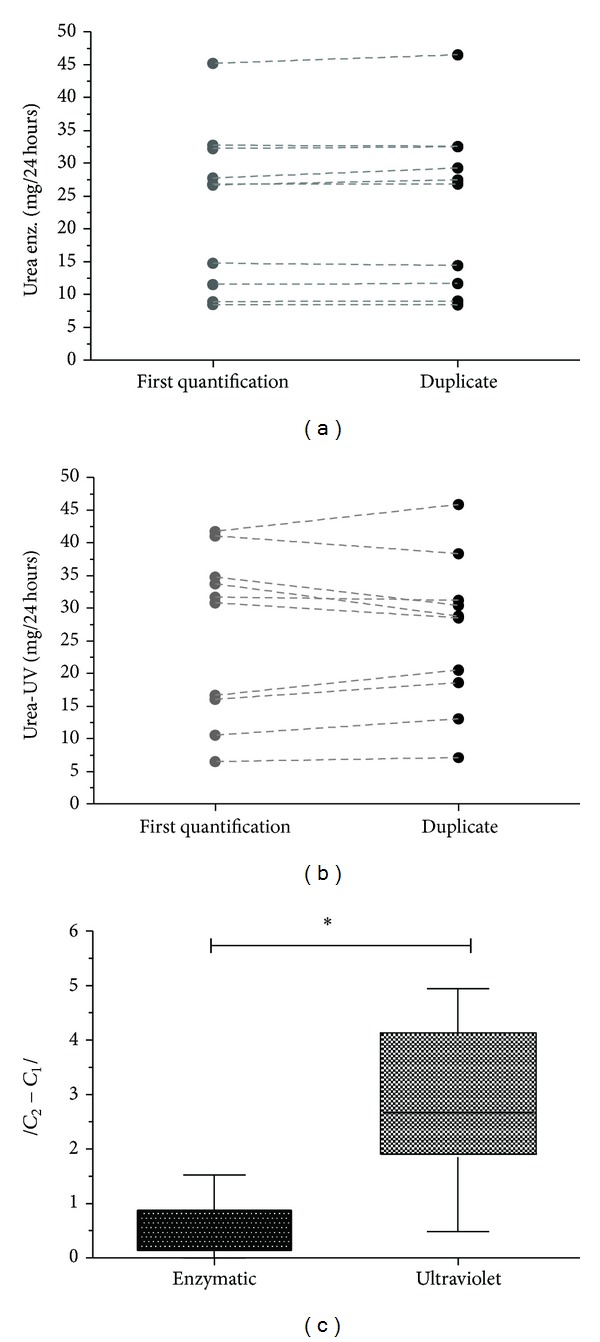
Methodological variation using duplicate samples. Urine was collected from C57BL/6 mice in metabolic cages for a period of 24 h. After centrifugation, the supernatant was removed for quantification of urea. The concentration of urea in the samples was quantified in duplicate, for each of two different methods, namely, the kinetic ultraviolet approach (kinetic reaction involving two time-points) and a colorimetric approach, using a fixed time period. (a) Assessment of the urea concentration in duplicate samples, measured by the enzymatic colorimetric method. (b) Assessment of the concentrations of urea in duplicate, measured by the ultraviolet method. (c) Evaluation of the differences between the duplicates by comparison of the two methodologies. The concentrations were expressed as mg per 24 h. Differences were considered significant when *P* < 0.05 (∗).

After defining the best means for storage, we proceeded to assess two different commercial kits (absorbance read at 580 or 340 nm) as well as the variations between their duplicates (1st and 2nd absorbance reading of the same sample, using the same kit). Our results showed no differences between the duplicates using the same kit for both the enzymatic urea determination methods (reading at 580 nm or 340 nm; Figure 4(a)). However, after subtraction of the two absorbance readings (duplicates), for comparison of the two methods, we found significant differences, with a greater variation in the ultraviolet-based method (reading at 340 nm; Figure 4(b)). Thus, duplicate measurements favor the accuracy of the evaluation of urea concentrations in the samples; but the ultraviolet method is more variable (Figure 4(c)).

## 4. Discussion

There is an increasing drive for researchers and laboratory services to guarantee accurate and reliable results in the analysis of patient as well as experimental animal samples [12]. When measuring kidney functional parameters, accurate assessment of biochemical changes in plasma and urine is essential. Yet, depending on the standard methodology, technique, origin, and quality of the samples used, the results may differ significantly, and therefore, measurements can be underestimated or overestimated [13]. In an attempt to reduce these variations, implementation of internal quality control procedures is crucial for ensuring accurate and reliable measurement of the analytes. Thus, choosing a good combination of decision criteria (control rules) to include in the quality control is essential for decreasing the inaccuracy of the analytical approach used.

In an attempt to better assess the renal function of mice, we here evaluated creatinine clearance, considering an average factor calculated from the BSA (determined from the weight and length of these animals) and used the Westgard rules [10] as a control strategy and the Levey-Jennings charts [14] to represent the data for each sample collection day. Although this approach has not been used much for murine samples, this strategy has already been adopted for reducing errors in the analysis of human and veterinary samples and has been used as an internal quality control for both a variety of samples and types of analyses [15–17].

As shown in Table 1, we evaluated the mean, standard deviation, and coefficient of variation in the length and weight of the mice, and from these data, we calculated the BSA and obtained an average factor of 0.006179. The results showed that when using the average factor to calculate creatinine clearance or urea, the values did not differ significantly. Thus, when we validated the data using the Westgard rules, none of the rules of quality control were rejected. Moreover, our data indicated that the urea and creatinine clearance measurements in mice should be adjusted to BSA as calculated based on the weight and length of these animals, to ensure that no systematic or random errors occur. Therefore, the results would be more precise and accurate than those evaluated without applying this internal quality control.

Concerning the effect of thawing on plasma urea concentration, we demonstrated that repeated freeze-thawing cycles affect the urea concentration in mouse samples. On the other hand, samples frozen at −20 or −70°C for 4 or 90 days, consecutively, showed no differences in the concentrations of plasma or blood urea. These results indicated that freeze-thawing cycles appear to be more problematic than long-term storage at −20 or −70°C in terms of sample maintenance.

The effects of freeze-thawing have been shown mainly in samples obtained from humans, nonhuman primates, and laboratory animals, such as rats and dogs [18–21]; however, controlled studies of the effects on mouse samples are in their infancy. According to Reynolds et al. [18], no clinically relevant changes are observed in different aliquots of canine plasma for all constituents, including urea and creatinine. Similar insignificant changes have been observed in cholesterol levels in baboon serum [22] and in cholesterol, micronutrients, and hormones in human plasma and serum samples [23]. However, Kale et al. [20] evaluated the effect of freeze-thawing on 18 different biochemical parameters of rat serum and showed that four of these were altered. In addition, a recent study also showed that after 90 days or 10 rounds of freeze-thawing, analytes such as glucose, creatinine, cholesterol, and triglycerides remain unchanged, but that concentrations of blood urea nitrogen, uric acid, lactate dehydrogenase, and so forth, changed significantly [21]. It is noteworthy that most of these studies had evaluated samples obtained from dogs, baboons, and humans only a few days after collection. In our case, the samples were evaluated within 90 days of collection, a storage period typical for research laboratories that need to collect a large number of samples before commencing biochemical evaluations. The fact that storage of our samples for 4 versus 90 days at −20 or −70°C did not result in significant differences in plasma or urine urea concentrations may suggest that the biggest problem arises from the repeated freezing and thawing of samples.

Thus, storage, freeze-thawing, and the use of internal quality controls are important steps in the preanalytical phase of laboratory testing and, depending on the implementation of these steps, the measurements of biochemical parameters may change considerably [21, 24].

With regard to variations between enzymatic and colorimetric methods for quantification of urea, we demonstrated that, although there were no differences between duplicate samples when using the same commercial kit, comparison of the subtracted values (between the two duplicates) revealed that the absorbance reading at 340 nm (ultraviolet method) presented greater variability in individual samples. Thus, our data indicated that the measurement of urea concentration is more reproducible when carried out by the enzymatic method.

## 5. Conclusions

Taken together, our results demonstrated that measurement of renal biochemical parameters in mice, such as urea and creatinine, can be measured with great accuracy when taking into consideration a factor calculated from the BSA. Moreover, repeated freeze-thawing cycles may induce important variations in the urea concentration. Additionally, the enzymatic method results in less variability in sample readings than does the ultraviolet method. Thus, we suggest that measurement of renal parameters need to be standardized and that samples, whether for laboratory testing or scientific research, must be appropriately handled prior to analysis.

## Figures and Tables

**Table 1:** Values for the calculation of body surface area (*n* = 40).

	*W* (kg)	*L* (cm)	*W* ^0.425^	*L* ^0.007184^	BSA
XM	0.021	7.79	0.20	4.43	0.006178561
Std. error	0.00026	0.056	0.00103	0.023	0.0000537
CV (%)	7.98	4.57	3.36	3.32	5.43

*W*: weight; *L*: length; BSA: body surface area; XM: mean; Std. error: standard error; CV: coefficient of variation.

**Table 2:** Data for the quantification of creatinine clearance (*n* = 9).

	PCr	Ucr	Uv	Ecc	CrCl∗0.006179
	mg/dL	mg per 24 h	mL/min	mL/min	mL/min
	6.15*E* − 01	4.71*E* − 01	3.48*E* − 03	5.32*E* − 02	4.61*E* − 02
	5.13*E* − 01	3.69*E* − 01	3.32*E* − 03	5.00*E* − 02	5.27*E* − 02
	3.08*E* − 01	2.22*E* − 01	4.87*E* − 03	5.01*E* − 02	4.99*E* − 02
	5.64*E* − 01	8.25*E* − 01	5.55*E* − 03	1.02*E* − 01	9.81*E* − 02
	4.10*E* − 01	4.43*E* − 01	4.08*E* − 03	7.50*E* − 02	7.44*E* − 02
	5.64*E* − 01	5.32*E* − 01	3.20*E* − 03	6.55*E* − 02	6.19*E* − 02
	9.74*E* − 01	2.67*E* − 01	1.19*E* − 03	1.90*E* − 02	2.01*E* − 02
	3.08*E* − 01	1.62*E* − 01	3.12*E* − 04	3.66*E* − 02	3.71*E* − 02
	4.62*E* − 01	3.10*E* − 01	9.72*E* − 04	4.66*E* − 02	4.66*E* − 02

Median	5.13*E* − 01	3.69*E* − 01	3.32*E* − 03	5.01*E* − 02	4.99*E* − 02
Maximum	9.74*E* − 01	8.25*E* − 01	5.55*E* − 03	1.02*E* − 01	9.81*E* − 02
Minimum	3.08*E* − 01	1.62*E* − 01	3.12*E* − 04	1.90*E* − 02	2.01*E* − 02

PCr: plasma creatinine; UCr: urinary creatinine; Uv: urinary volume; Ecc: endogenous creatinine clearance; CrCl: creatinine clearance.
